# A Genome-Wide Association Study Reveals New Loci for Resistance to Clubroot Disease in *Brassica napus*

**DOI:** 10.3389/fpls.2016.01483

**Published:** 2016-09-30

**Authors:** Lixia Li, Yujie Luo, Biyun Chen, Kun Xu, Fugui Zhang, Hao Li, Qian Huang, Xin Xiao, Tianyao Zhang, Jihong Hu, Feng Li, Xiaoming Wu

**Affiliations:** Key Laboratory of Biology and Genetic Improvement of Oil Crops, Ministry of Agriculture, Oil Crop Research Institute – Chinese Academy of Agricultural SciencesWuhan, China

**Keywords:** *Brassica napus* L., *Plasmodiophora brassicae*, clubroot resistance, genome wide association study, candidate gene prediction

## Abstract

Rapeseed (*Brassica napus* L.) is one of the most important oil crops in the world. However, the yield and quality of rapeseed were largely decreased by clubroot (*Plasmodiophora brassicae* Woronin). Therefore, it is of great importance for screening more resistant germplasms or genes and improving the resistance to *P. brassicae* in rapeseed breeding. In this study, a massive resistant identification for a natural global population was conducted in two environments with race/pathotype 4 of *P. brassicae* which was the most predominant in China, and a wide range of phenotypic variation was found in the population. In addition, a genome-wide association study of 472 accessions for clubroot resistance (CR) was performed with 60K *Brassica* Infinium SNP arrays for the first time. In total, nine QTLs were detected, seven of which were novel through integrative analysis. Furthermore, additive effects in genetic control of CR in rapeseed among the above loci were found. By bioinformatic analyses, the candidate genes of these loci were predicted, which indicated that TIR-NBS gene family might play an important role in CR. It is believable that the results presented in our study could provide valuable information for understanding the genetic mechanism and molecular regulation of CR.

## Introduction

Rapeseed (*Brassica napus* L., AACC, 2*n* = 38) is one of the most important and widely cultivated oil crops, which derived from the hybridization of two basic diploid species in U-triangle, *Brassica rapa* (AA, 2*n* = 20) and *Brassica oleracea* (CC, 2*n* = 18; Nagaharu, 1935). The completion of genome sequencing of A genome from *B. rapa* (Wang et al., 2011), C genome from *B. oleracea* (Liu et al., 2014) and AC genome from *B. napus* (Chalhoub et al., 2014) could provide crucial information for studying the genetic and molecular mechanisms of important traits.

Clubroot, caused by *Plasmodiophora brassicae* Woronin, is an obligate and devastating disease. The pathogen could infect about 3,700 species through 330 genera in Brassicaceae (Hwang et al., 2012), among that the host range is most widespread in *Brassica, Raphanus*, and *Arabidopsis* (Dixon, 2009). Physiological specialization has long been known to occur in *P. brassicae* (Honig, 1931). The Williams classification (Williams, 1966) and European Clubroot Differential (Buczacki et al., 1975) have been used commonly for assessment of virulence in *P. brassicae*. In China, race/pathotype 1, 2, 4, 7, 9, 10, 11, and 13, classified on the differentials of Williams, have been identified. Furthermore, race/pathotype 4 was found predominant in China (Chai et al., 2014). After infected by *P. brassicae*, the root of host became proliferation and deformity, leading to the formation of typical clubs (Dobson and Gabrielson, 1983). The symptoms prevented the root cells from absorption water and nutrition, and caused the host plant malnutrition, growth retardation even death, which resulted in reduction of crop production and quality. Studies showed that there was a reduction of 30%, even to 80–91% for yield loss in rapeseed experimental field trials (Tewari et al., 2005; Pageau et al., 2006). Aside from production, the seed quality was also decreased with a loss of 4.7–6.1% in oil content, 13–26% in 1000 seed weight (Hwang et al., 2012), and an increase of 50% in oil chlorophyll content (Engqvist, 1994). Although various managements have been used to control the clubroot (Kowata-Dresch and May-De Mio, 2012), improving varieties with durable resistance by classical breeding or genetic modification was still an effective and environment-friendly way.

Sources of resistance to *P. brassicae* have been identified in *Brassica* germplasms. Among the European fodder turnips (*B. rapa* ssp. *rapifera*), several clubroot-resistant cultivars including Gelria R, Siloga, Debra, and Milan White were found (Hirai, 2006). In contrast to *B. rapa*, the immune resources have only rarely been identified in *B. oleracea* and *B. napus* (Piao et al., 2009). Nevertheless, some high-resistant resources were also used to develop clubroot resistance (CR) cultivars with diverse resistant backgrounds in *B. napus* (Rahman et al., 2011).

Genetic mapping of CR has been performed extensively on resistant resources. In *Arabidopsis thaliana*, a CR gene *RPB1*, four additive QTLs, and four epistatic regions were identified (Fuchs and Sacristán, 1996; Jubault et al., 2008). Most of the known CR genes/QTLs were identified from *B. rapa*. At least 11 CR genes/QTLs were mapped on five different chromosomes based on different bi-parental linkage population, which were *Crr2* on BrA01(Suwabe et al., 2003), *Crr3* and *CRc* on BrA02 (Hirai et al., 2004; Sakamoto et al., 2008), *CRa, CRb, CRk, PbBa3.1, PbBa3.3*, and *Rcr1* on BrA03 (Matsumoto et al., 1998; Piao et al., 2004; Sakamoto et al., 2008; Kato et al., 2012; Chen et al., 2013; Chu et al., 2014), *Crr4* on BrA06 (Suwabe et al., 2006), *Crr1* on BrA08 (Suwabe et al., 2003). To increase efficiency of marker assisted selection (MAS), fine mapping was carried out for *CRb, Crr3*, and *Rcr1* (Saito et al., 2006; Kato et al., 2013; Chu et al., 2014; Zhang et al., 2014). In contrast to *B. rapa*, fewer CR loci, such as *CR2a, CR2b, Pb3, Pb4, PbBo1, Pb-Bo* (Anju 1), *CRQTL-YC, CRQTL-GN_1*, and *CRQTL-GN_2* were identified in *B. oleracea* (Landry et al., 1992; Grandclément and Thomas, 1996; Voorrips et al., 1997; Rocherieux et al., 2004; Nagaoka et al., 2010; Lee et al., 2016). In *B. napus*, one dominant gene *Pb-Bn1*, and two other QTLs were mapped on the BnA04 and BnC05, respectively (Manzanares-Dauleux et al., 2000). As well as 19 QTLs were detected on eight chromosomes (Werner et al., 2008). One locus in a population of 121 doubled haploid lines was identified to be linked to *CRa* which was identified in *B. rapa* (Zhang et al., 2015).

Although, QTL mapping based on bi-parental population has been used in the study of genetic architecture of complex traits (Cai et al., 2016), it was also restricted for the limited recombination events between parents and the difficult-used marker information in previous study. With the development of high-throughput sequencing technology, genome-wide association study (GWAS) based on linkage disequilibrium (LD) has become an important and powerful tool for gene mapping. GWAS could take advantage of the phenotypic variation and historical recombination in natural populations without constructing a mapping population (Nordborg and Weigel, 2008). In recent years, GWAS has been used to identify genes of various traits in rapeseed, such as traits of yield, seed quality, flowering time, and resistance to *Sclerotinia sclerotiorum* (Harper et al., 2012; Lu et al., 2014; Raman et al., 2014; Li et al., 2015; Liu J. et al., 2016; Liu S. et al., 2016; Wei et al., 2016; Xu et al., 2016). However, there was no report of GWAS being used to study resistance to *P. brassicae* in *Brassica*.

Plants have evolved two innate immune pathways to resist the attack of pathogens (Jones and Dangl, 2006). The first one was pathogen-associated molecular pattern (PAMP) triggered immunity (PTI), which the pattern recognition receptor (PRR) proteins located on the external face of the host cell could recognize the conservative PAMPs released by pathogens, sequentially activating the PTI regulated by multiple signal transduction pathway; The other one was effector-triggered immunity (ETI), which the effector factor secreted by pathogens could inhibit the PTI, and be recognized by specific *R* genes in plants, consequently triggering the ETI. With the action of natural selection, pathogens would evolve new effectors, and plants also evolved new *R* genes (Bent and Mackey, 2007). Most R proteins contain conserved motifs such as nucleotide-binding site (NBS), leucine-rich repeat (LRR), Toll-interleukin-1 receptor domain (TIR), coiled-coil (CC) or leucine zipper (LZ) structure and protein kinase domain (PK, Liu et al., 2007). Among the identified CR loci, *CRa* and *Crr1* have been cloned and confirmed to carry TIR-NBS-LRR structure (Ueno et al., 2012; Hatakeyama et al., 2013).

In this study, a massive resistant identification for a natural global population including 472 accessions was conducted with pathotype 4 of *P. brassicae* which was the most predominant in China. Based on the phenotypic data, a GWAS of 472 accessions was performed in CR with *Brassica* 60K SNP arrays at two environments for the first time. The aim of this study was to gain resistant resources from rapeseed by identification in multiple environments, and to detect CR loci. Some candidate genes were predicted by bioinformatic analyses. These findings would provide valuable information for understanding the genetic mechanism and molecular regulation of CR, and also for resistant breeding to *P. brassicae* in *Brassica*.

## Materials and Methods

### Plant Materials and Pathogen Isolates

The association population of rapeseed including 472 accessions, which were collected from 23 countries worldwide of four continents, has been previously reported (Li et al., 2014). All the seeds of these inbred lines were conserved in the National Mid-term Gene Bank for Oil Crops in Wuhan, China. To carry out the artificial inoculation in greenhouse (GH), the infected roots of rapeseed were collected from the infected field (IF) of Dangyang, China, where the pathogen was reported as pathotype 4 based on Williams classification (Ren et al., 2012).

### Field Experiments and Artificial Inoculation

All of the 472 accessions were planted in field infected seriously by *P. brassicae* at Dangyang, China on October 2013. The field experiments followed a randomized complete block design with two rows and three replications. Before sowing, we partitioned compartments with width of 2.0 m in consideration of field drainage and soil fertility factors. The plants were sown in strip with a 0.3 m spacing between rows, singling 12 plants each row after sowing for 1 month. Infected rapeseed roots collected from Dangyang were stored at -20°C, which were used for artificial inoculation trials in GH. The artificial inoculation of the population was performed at Shenyang on October of 2015. Firstly, nutrient soil was dried and powdered, while the disease roots were unfrozen and decomposed for one night. Then we squeezed the disease roots with distilled water to make *P. brassicae* suspension. The suspension was mixed with the dried nutrient soil and adjusted to 10^8^ resting spores per gram (g) dry soil. The soil humidity which reached to the extent of kneading with clumps and touching with scatter was advisable, and then was sealed to ferment for 2 days in dark. The hole tray was filled with nutritional soil and then irrigated enough water. About 3 g fermentative soil per hole was placed, and sowed two seeds in the fermentative soil. The artificial inoculation followed a randomized complete block design with three replications, and inoculated 24 seeds for each replication. The plants grew under a 16 h L/8 h D photoperiod at an average temperature of 20–25°C.

### Evaluation of Clubroot Reaction, the Broad-Sense Heritability, and Statistical Analysis

At flowering time of IF trials and 50 days after inoculation in greenhouse, plants were all taken out with roots and the disease severity was assessed using a 0–3 scale as follows, 0 = no clubs, 1 = a few small clubs, 2 = moderate clubbing, and 3 = severe clubbing (Kuginuki et al., 1999). A disease index (DI) was calculated according to the formula DI = [(*n*_1_+2*n*_2_+3*n*_3_)/(*N*_T_ × 3)] × 100, where *n*_1_ to *n*_3_ were the number of plants with different disease severity of 1–3 scale and *N*_T_ represented the total number of identified plants, respectively. An incidence rate (IR) was calculated with a formula IR (%) = [(*n*_1_+*n*_2_+*n*_3_)/*N*_T_] × 100. The broad-sense heritability was calculated as *h^2^*= σ^2^_g_/(σ^2^_g_+σ^2^_ge_/*n*+σ^2^_e_/*nr*). Where σ^2^_g_ is the genetic variance, σ^2^_ge_ is the interaction variance of the genotype with environment, σ^2^_e_ is the error variance, *n* is the number of environments and *r* is the number of replications. The estimates of σ^2^_g_, σ^2^_ge_, and σ^2^_e_ were obtained from the analysis of variance (ANOVA) procedure in SPASS 20.0 and the frequency distribution of phenotype was carried out using Excel 2010. The multiple comparisons of phenotypic average value from different origins were tested with package of SAS 9.2 at a significance level of *P* < 0.05. Also, the difference analysis of genotypic effects with containing different No. of favorable SNPs was performed online^1^.

### SNP Array Analysis and Mapping, Population Structure and Linkage Disequilibrium Analysis

The young leaves from the representative plants of all accessions were collected, and extracted their genomic DNAs using a modified cetyltrimethyl ammonium bromide (CTAB, Murray and Thompson, 1980) method. SNP genotyping of 472 accessions was performed using *Brassica* 60K Illumina Infinium^®^ HD Assay SNP arrays by Emei Tongde, Co. (Beijing) according to the manufacturer’s protocol^2^. The quality control process of SNP array genotyping data was the same to our previous study (Li et al., 2014). The LD was calculated using the software TASSEL3.0 (Bradbury et al., 2007). The principal component analysis (PCA), population structure and kinship were analyzed by Li et al. (2014). To confirm the physical position of each SNP, the probe sequences of 26,841 high-quality SNPs previously selected (Li et al., 2014) were used to perform a BlastN search against the *B. napus* (*Darmor-bzh*) reference genome by Li et al. (2016). Only the top blast hits were considered to be mapped in the genome with an *E*-value threshold of 1E–15, and blast matches to multiple loci with the same top *E*-value were not considered to be mapped successfully. Totally, 19,945 SNPs were assigned to *B. napus* chromosomes.

### Genome-Wide Association Study

To evaluate the effects of population structure (Q, PC), the trait-SNP association analysis was performed using TASSEL 4.0 with six models: (i) GLM, controlling for nothing, (ii) Q model, controlling for Q, (iii) PCA model, controlling for PC, (iv) K model, controlling for K, (v) Q+K model, controlling for both Q and K, (vi) P+K model, controlling for both PC and K. The GLM, Q, K, and PCA models were performed using a general linear model with the following equation: y = Xα+ e. The Q+K and P+K models were performed using a mixed linear model with the following equation: y = Xα+Kμ+e. In above equations, y represented phenotype, X represented genotype, α was a vector containing fixed effects, K was the relative kinship matrix, μ was a vector of random additive genetic effects, and e was the unobserved vector of random residual. The distribution of observed -log_10_(p) for each SNP from marker-trait associations was compared with the expected distribution in a quantile-quantile plot (Q-Q plot). The significance of associations between SNPs and traits was based on a threshold *P* < 5.01 × 10^-5^ [P = 1/N, where N = the number of SNPs used, -log_10_(1/19,945) = 4.3]. Meanwhile, to avoid ignoring the effects of minor loci, the threshold P was lowered to 2/N = 1.00 × 10^-4^. Q-Q plots and Manhattan plots were drawn using the R package qqman^3^.

### QTL Alignment and Candidate Gene Prediction

To compare the QTLs detected in this study with previous studies on CR, the sequences of QTLs/genes which have been reported in previous studies were collected and performed BlastN or e-PCR on the *B. napus* reference genome (*Darmor-bzh*) with a threshold of 1E–10. By that, we could ensure the homoeologous fragments’ physical positions of QTLs/genes reported in previous studies on *B. napus* genome.

It was regarded as a LD block that the region containing all SNPs which *r*^2^ > 0.4 with the most significant SNP. To ascertain regions that potential candidate genes of each QTL identified in this study maybe located, we considered the flanking markers that outside and adjacent to the LD block were considered as the candidate region’s boundary. To further identify the candidate genes and the participated pathways of the identified loci, the gene function annotation and enrichment analysis were carried out. All *B. napus* genes which located in the candidate regions were searched against the NCBI non-redundant (Nr) protein database using BlastP with an *E*-value ≤ 1E–05. The gene ontology (GO) terms of the *B. napus* genes were annotated by merging the Blast2GO and InterPro annotation results (Wu et al., 2016). GO enrichment analysis provided all of the GO terms that were significantly enriched in candidate genes compared with the genome background using Blast2GO with a false discovery rate (FDR) ≤ 0.01. The published differential expressed genes (DEGs) information in *B. rapa* were downloaded (Chen et al., 2015), and searched the homoeologous genes in *B. napus* with a threshold of 1E–50.

## Results

### Phenotypic Variation of CR Revealed its Genetic Complexity in Rapeseed

The 472 rapeseed accessions were planted in two environments of IF in Dangyang at the year of 2013–2014, and GH in Shenyang at the year of 2015, respectively. DI and IR were used to evaluate extent of CR. The frequency distribution diagrams of these two indicators in the two environments all showed continuous distributions (Supplementary Figure S1), which indicated that DI and IR were both controlled by multiple loci. Extensive variations of these two indicators were observed among the population in both two environments (**Table 1**). In IF environment, DI ranged from 9.26 to 75.00, with an average value of 31.39 ± 5.92 (± SE); IR ranged from 18.18 to 100% with an average value of 48.11 ± 8.10% (± SE). The broad-sense heritability (*h^2^*) of DI and IR in IF were 64.8 and 55.0%, respectively (**Table 1**). In GH environment, DI was from 36.11 to 100.00, with an average value of 84.77 ± 9.67 (±SE); IR was from 58.26 to 100%, with an average value of 96.49 ± 7.64% (± SE). Moreover, the *h^2^* of DI and IR in GH were 78.2 and 69.4%, respectively (**Table 1**). The results thus indicated that the stability of CR is high, especially in a control environment. The correlation analysis of these two indicators between the two environments showed that the correlation reached to highly significant level, but the value of which (0.24 and 0.19) is too low (Supplementary Table S1). The results indicated that genetic factor and environment factor both played an important role in CR.

**Table 1:** The performance and heritability of clubroot resistance of the natural population in *Brassica napus*.

Environment	Trait	Minimum	Maximum	Mean ± SE	*h^2^* (%)
Infected field	DI	9.3	75.0	31.39 ± 5.92	64.8
	IR	18.2	100.0	48.11 ± 8.70	55.0
Green house	DI	36.1	100.0	84.77 ± 9.67	78.2
	IR	58.3	100.0	96.49 ± 7.64	69.4

*DI, disease index; IR, incidence rate (%); *h^*2*^*, broad-sense heritability.*

To assess the effects of origin including different continent (Europe, Asia, America, and Oceanica), different country (China and abroad), ecotype (spring, semi-winter, and winter type), and breeding eras in China (the years of 1950–1970, 1970–1980, 1980–1990, 1990–2000, and 2000–2011) on CR in the population, the accessions were classified by the above categories, respectively (Supplementary Figure S2). The DI and IR were both compared among different categories, and the results showed that the accessions from China were more resistant than that of from abroad both in DI and IR of two environments. Nevertheless, the absolute difference of these two indictors between China and abroad were very small (Supplementary Figure S2A). The similar tendency was emerged in the comparison among Europe, Asia, America, and Oceanica. The accessions from Asia and Europe were more resistant (Supplementary Figure S2B). Moreover, the semi-winter ecotype accession was the most resistant among the different ecotypes (Supplementary Figure S2C). The resistance of accessions from different breeding eras had significant difference (Supplementary Figure S2D), which indicated that artificial selection could impact the CR significantly. Therefore, we can further reinforce the resistance breeding in *B. napus*.

### Genome-Wide Association Mapping Identified More Loci for CR in *B. napus*

In order to select suitable model for association mapping, GWAS was performed using four general liner models (GLM, Q, K, and P) and two mixed liner models (Q+K, and P+K) for IF-DI (DI in IF), IF-IR (IR in IF), GH-DI (DI in GH), and GH-IR (IR in GH), respectively. As the Q-Q plot showed, the distribution of observed -log_10_(p) from P+K model was the closest to the expected distribution for all four traits, which led to a low level of false-positive signals (Supplementary Figure S3). Therefore, the association signals were identified with P+K model subsequently.

**FIGURE 1:**
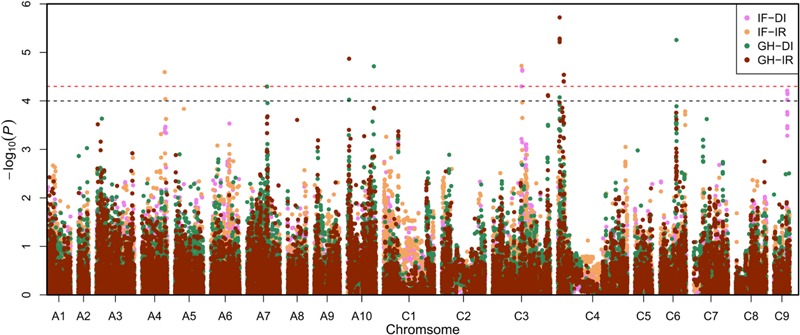
**Manhattan plots of association analysis using the mixed liner model (MLM) model P+K.** The pink plots, yellow plots, green plots and brown plots represent the associated signals for infected field-disease index (IF-DI), infected field-incidence rate (IF-IR), greenhouse-disease index (GH-DI), and greenhouse-incidence rate (GH-IR), respectively. The black and red dashed horizontal line depict the two significant thresholds that is (-log_10_1/19,945 = 4.30) and (-log_10_2/19,945 = 4.00).

To avoid ignoring the micro effective locus, significant associated SNP (sSNP, *P <* 5.01 × 10^-5^) and potential association SNP (pSNP, 5.01 × 10^-5^ < *P* < 1.00 × 10^-4^) were introduced in the study. If a confidence interval of a locus included one or more sSNPs, we called it significant association locus. Similarly, if a confidence interval of a locus did not have sSNP, and only included two or more pSNPs, we called it potential association locus. In total, seven significant association loci and two potential association loci were identified with these two indicators (**Figure 1**).

For IF-DI, one significant association locus on BnC03 (named *MCR-C3*) was identified, with a peak SNP (highest significant) Bn-scaff_17521_1-p419499 which contributed to 4.81% of phenotypic variance. One potential association locus on BnC09 (named *MCR-C9*) was detected, with a peak SNP Bn-scaff_15576_1-p660538 which explained 4.21% of phenotypic variance (**Table 2**). For IF-IR, one significant association locus on BnA04 (named *MCR-A04*) was mapped, with a peak SNP Bn-A04-p16156157 which contributed 4.72% to phenotypic variance. It was noteworthy that *MCR-C3* in IF-DI was also detected in IF-IR. For GH-DI, one significant association locus *SCR-C6* was detected on BnC06 with a peak SNP Bn-scaff_16064_1-p26703 which explained 5.26% of phenotypic variance. Another significant association locus *SCR-A10a* was identified on BnA10 with a peak SNP Bn-A10-p16087066 which accounted for 4.71% of phenotypic variance. For GH-IR, three significant association loci were identified in total. Among that two loci were both on BnC04 (named *SCR-C4a* and *SCR-C4b*), which explained 5.72 and 4.54% of phenotypic variance, respectively. The rest one named *SCR-A10b* was on BnA10, with a peak SNP Bn-A10-p3966740 which explained 4.87% of phenotypic variance. In addition, one potential association locus *SCR-C3* was mapped on BnC03 with a peak SNP Bn-scaff_18559_1-p166394 which accounted for 4.12% of phenotypic variance (**Table 2**).

**Table 2:** The identified QTLs information through GWAS analysis in the natural population of *B. napus.*

Trait	Locus^a^	Chr	SNP No.^b^	MSS-R^2^(%)^c^	Position range (bp)
IF-DI	*MCR-C3*	C03	4	4.81	21,372,298–21,810,339
	*MCR-C9*	C09	0 (8)	4.21	41,753,926–41,967,651
IF-IR	*MCR-A4*	A04	1 (1)	4.96	16,356,482–16,733,484
	*MCR-C3*	C03	1	4.88	21,372,298
GH-DI	*SCR-A10a*	A10	1	4.96	15,476,654
	*SCR-C6*	C06	1	5.9	25,596,721
GH-IR	*SCR-A10b*	A10	1	5.19	885,083
	*SCR-C3*	C03	0 (6)	4.34	58,088,063–58,097,249
	*SCR-C4a*	C04	4	6.51	2,498,886–2,511,207
	*SCR-C4b*	C04	8	4.79	8,065,329–8,102,210

*^a^QTL name identified in this study; MCR: QTL identified in infected field; SCR: QTL identified in green house. ^b^The number out of bracket depict the No. of significant associated SNP and the number in bracket represent the No. of potential associated SNP. ^c^Percentage of phenotypic variance explained by that of peak SNP marker.*

### Combined Effects Analysis Exhibited Additive Effects on CR

**FIGURE 2:**
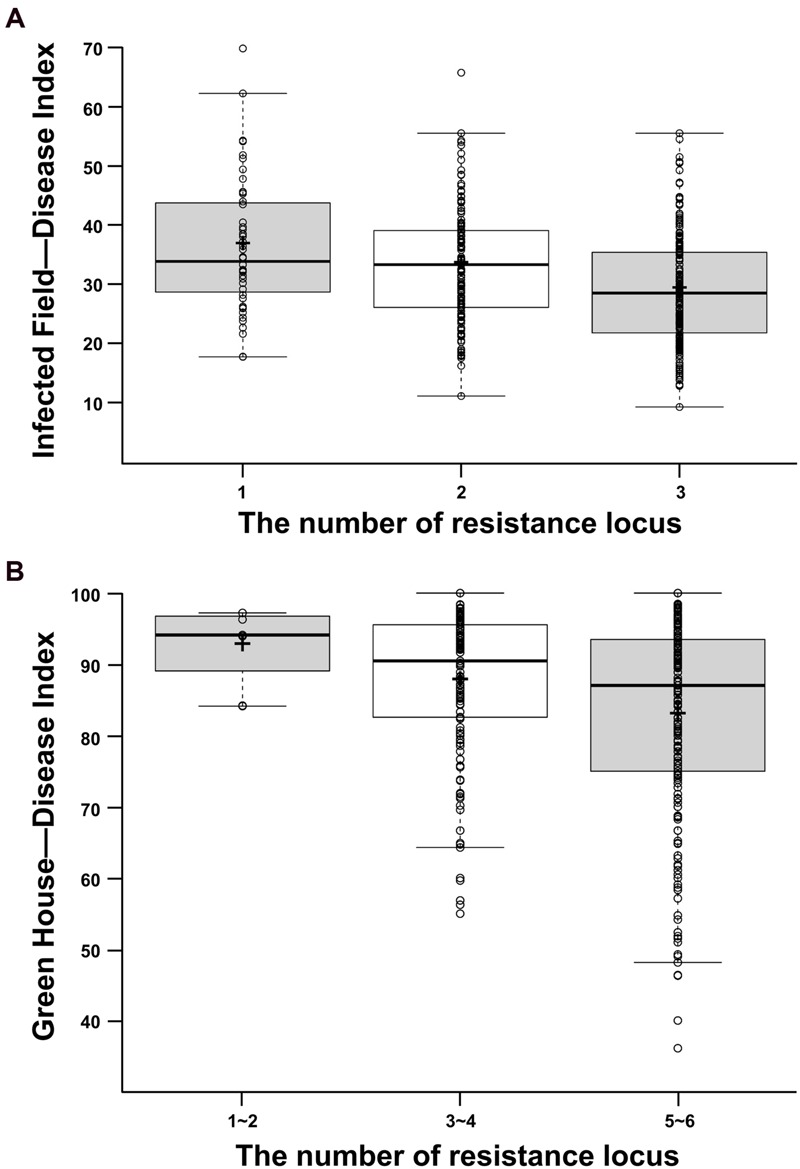
**Box plot for DI in two environments. (A)** Box plots for DI in IF. **(B)** Box plots for DI in GH. The middle line indicates the median, the plus sign shows the mean, the box represents the range of the 25th to 75th percentiles of the total data, the whiskers shows the interquartile range, the two line segments outside of the box indicates the boundary of normal value and the out dots means the outliers.

To understand the effects of allelic variations on CR in rapeseed, the combined effects of DI of different QTLs were analyzed in IF and GH, respectively (**Figure 2**). The genotype and phenotypic variance (*R^2^*) of each QTL was substituted by that of each peak marker of the corresponding QTL. On this basis, 472 accessions were grouped into three classes which contained 1, 2, and 3 favorable alleles in IF, respectively (**Figure 2A**). Because of too less accession contained in one group if all accessions were grouped by containing 1–6 favorable alleles in GH, all these six loci were merged and divided into three groups which contained 1–2, 3–4, and 5–6 favorable alleles to statistical analyze the DI in GH (**Figure 2B**). The results indicated that the more favorable alleles (resistant alleles) had, the lower DI (more resistance to *P. brassicae*) was demonstrated (**Figure 2**). In IF, the DI of the accessions held three resistant alleles can be reduced by 20.62% compared with that of holding one resistant allele. Similarly, the DI of the accessions held 5–6 resistant alleles can be reduced by 10.56% compared with that of only holding 1–2 resistant alleles in GH. The above results revealed that the CR was mainly controlled by additive effect. Therefore, the CR for *B. napus* can be improved by polymerization of all identified resistant alleles.

### Integrative Analysis of CR Loci/Genes Revealed the Novel Loci Detected in this Study

**FIGURE 3:**
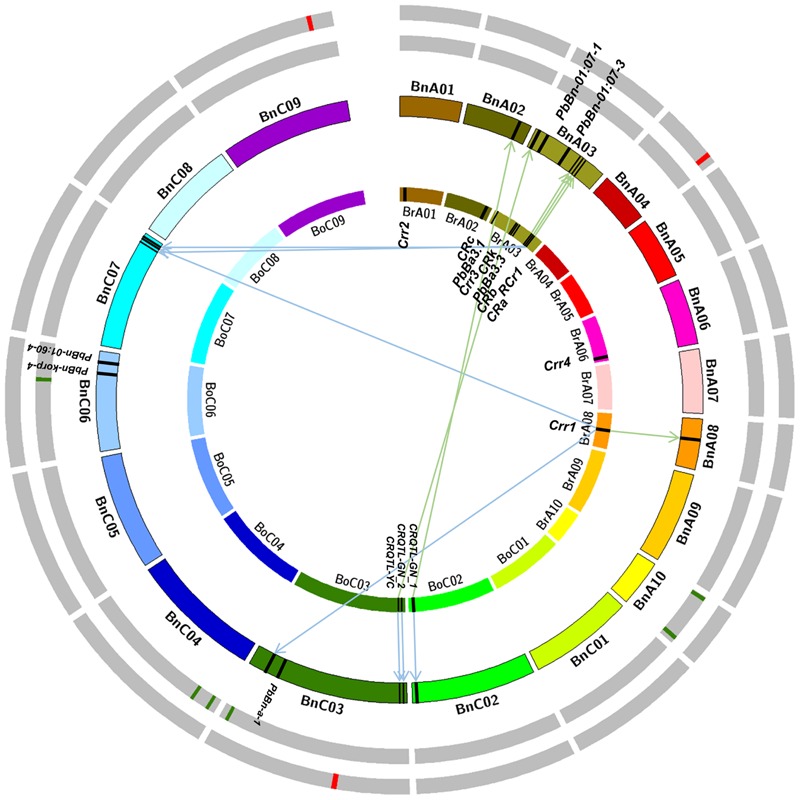
**Integrative physical map of clubroot resistance (CR) sites based on the mapping results of the *Brassica rapa, Brassica Oleracea*, and *Brassica napus*.** The right hand side of the innermost circle depicts the 10 chromosomes of *B. rapa*, the black square on that represents the CR loci identified in *B. rapa*; The left hand side of the innermost circle depicts the nine chromosomes of *B. oleracea*, the black squares on that represents the CR loci identified in *B. oleracea*. The second circle (from the innermost one) depicts the 19 chromosomes of *B. napus*, the black squares on that represents the CR loci which identified in previous reports in *B. napus* (without arrow) or the homoeologous regions (with arrow) of the black squares on the innermost circle. The green squares on the third circle represent the CR loci identified in greenhouse in this study. The red squares on the outermost circle represent the CR loci identified in infected field in this study.

To compare the GWAS results in this study with the previous reported CR QTLs/genes, the sequences of CR genes and markers of QTLs related to CR were collected in *B. napus, B. rapa*, and *B. oleracea*. And then the blastn or e-PCR with a threshold of 1E–10 was performed to *B. napus* reference genome (*Darmor-bzh*) to search their homoeologous regions in *B. napus* (**Figure 3**). In *B. rapa*, two homoeologous regions of *CRa* were identified on BnA03 (22,864,716–22,877,171 bp) and BnC07 (38,863,628–38,883,622 bp), respectively (**Figure 3**). Three homoeologous regions of *Crr1* were found on BnA08 (9,456,084–9,467,947 bp), BnC03 (54,084,701–54,109,095), and BnC07 (38,863,628–38,869,983 bp), respectively (**Figure 3**). One homoeologous region of *CRb* was identified on BnA03 (21,682,961–22,238,961 bp). Two homoeologous regions of *Rcr1* were found on BnA03 (22,797,049–23,009,129 bp) and BnC07 (36,229,328–39,036,510 bp) in *B. napus*. Homoeologous regions of other QTLs in *B. rapa* were not identified, because of their limited marker information or the physical positions of forward and reverse markers linked with the QTLs were not on the same chromosome in *B. napus* genome. Because of the limited reports on CR studies in *B. oleracea*, only three QTLs *CRQTL-YC, CRQTL-GN_1*, and *CRQTL-GN_2* were searched. Two homoeologous regions of *CRQTL-YC* were identified on BnA03 and BnC03 respectively. Also, two homoeologous regions of *CRQTL-GN_1* were detected on BnA02 (22,576,577–22,970,418 bp) and BnC02 (42,737,365–43,546,906 bp). And one homoeologous region of *CRQTL-GN_2* was mapped on BnC03 (1,185,066–2,835,468 bp; **Figure 3**).

Moreover, it was attempted to ensure the QTL physical position in *B. napus* according to the providing markers information in previous reports. Markers sequences of five QTLs reported on previous study in *B. napus* were searched and the physical position of *PbBn-01:07-1* and *PbBn-korp-4* were confirmed successfully. The physical positions of these two QTLs, BnA03 (5,386,730–5,386,845 bp) and BnC06 (27,067,129–27,067,246 bp), were proved to be accordance with the previous reports. However, the physical positions of other three QTLs were not accordance with the previous studies. For example, the QTL *PbBn-a-1* located on BnA08 was mapped on BnC03, and the *PbBn-01:07-3* located on BnC03 was mapped on BnA03 in this study (**Figure 3**).

It was found that the three QTLs identified in IF in our study were all novel, in which regions there were no homoeologous regions of CR QTLs reported in previous studies (**Figure 3**). The QTL *SCR-C3* identified in GH was not located on the confidence intervals of one homoeolog of *Crr1*, but was close to it. We considered it as an old CR locus. The same situation was on *SCR-C6*, which closed to the physical position of *PbBn-korp-4*. The other four QTLs identified in this study were novel. So the GWAS had stronger ability to detect new loci on a given trait.

### Prediction of the Candidate Genes of Identified CR QTLs by Bioinformatic Analyses

To predict the candidate genes of CR QTLs identified in this study, the candidate region (confidence interval) for each QTL was confirmed. There were 542 predicted genes in all nine candidate regions, 471 genes of which had functional annotations in total. To gain insights into the functionality of above genes, we performed GO enrichment analysis using Blast2GO (Conesa et al., 2005). The result showed that eight pathways of the molecular function related to ADP binding and methylthiopropyl-desulfoglucosinolate sulfotransferase activity were enriched (Supplementary Figure S4), the pathways of which were highly significant with TIR-NBS-LRR genes. It was interesting that two TIR-NBS gene clusters held 28 TIR-NBS genes which located in *SCR-C6* (10 TIR-NBS genes) and *MCR-C9* (18 TIR-NBS genes; **Table 3**) were participated in the function of ADP binding. The results indicated that the TIR-NBS gene family may associated with CR.

**Table 3:** The details of the TIR-NBS gene clusters in the candidate gene regions of *SCR-C6* and *MCR-C9*.

Locus	Candidate gene region (Mb)	TIR-NBS gene cluster	Description
*SCR-C6*	25.09–26.22	BnaC06g23900D	Disease resistance protein (TIR-NBS class)
		BnaC06g23910D	Disease resistance protein (TIR-NBS class)
		BnaC06g23920D	Disease resistance protein (TIR-NBS class)
		BnaC06g23930D	Disease resistance protein (TIR-NBS class)
		BnaC06g23940D	Disease resistance protein (TIR-NBS-LRR class)
		BnaC06g23950D	Disease resistance protein (TIR-NBS-LRR class) family
		BnaC06g23970D	Disease resistance protein (TIR-NBS class)
		BnaC06g23980D	Disease resistance protein (TIR-NBS-LRR class) family
		BnaC06g24000D	Disease resistance protein (TIR-NBS class)
		BnaC06g24010D	Disease resistance protein (TIR-NBS-LRR class)
*MCR-C9*	41.72–42.80	BnaC09g39420D	Disease resistance protein (TIR-NBS-LRR class), putative
		BnaC09g39430D	Disease resistance protein (TIR-NBS-LRR class) family
		BnaC09g39440D	Disease resistance protein (TIR-NBS-LRR class) family
		BnaC09g39450D	Disease resistance protein (TIR-NBS-LRR class) family
		BnaC09g39460D	Disease resistance protein (TIR-NBS-LRR class) family
		BnaC09g39470D	Disease resistance protein (TIR-NBS-LRR class) family
		BnaC09g39490D	Disease resistance protein (TIR-NBS-LRR class) family
		BnaC09g39500D	Disease resistance protein (TIR-NBS-LRR class) family
		BnaC09g39520D	Disease resistance protein (TIR-NBS-LRR class) family
		BnaC09g39560D	Disease resistance protein (TIR-NBS-LRR class) family
		BnaC09g39570D	Disease resistance protein (TIR-NBS-LRR class) family
		BnaC09g39590D	Disease resistance protein (TIR-NBS-LRR class) family
		BnaC09g39630D	Disease resistance protein (TIR-NBS-LRR class) family
		BnaC09g39890D	Disease resistance protein (TIR-NBS-LRR class) family
		BnaC09g39900D	Disease resistance protein (TIR-NBS-LRR class) family
		BnaC09g40030D	Disease resistance protein (TIR-NBS-LRR class)
		BnaC09g40060D	Disease resistance protein (TIR-NBS-LRR class)
		BnaC09g40250D	Disease resistance protein (TIR-NBS-LRR class), putative

**FIGURE 4:**
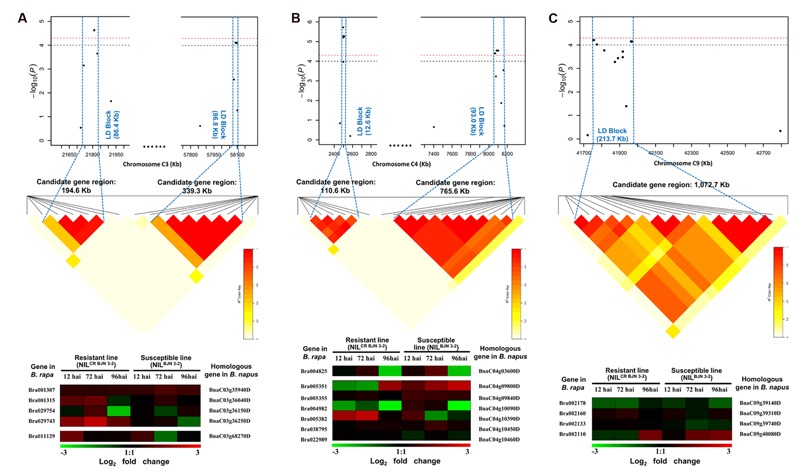
**The candidate regions and predicting candidate genes for part of quantitative trait loci (QTL) identified in this study. (A)** The candidate regions and predicting candidate genes for *MCR-C3* and *SCR-C3.* **(B)** The candidate regions and predicting candidate genes for *SCR-C4a* and *SCR-C4b.* **(C)** The candidate regions and predicting candidate genes for *MCR-C9*. Haplotype block in strong LD (*r*^2^ > 0.4) with the most significant associated SNPs are shown between the blue dashed line. The chromosome region between the two flanking markers of the LD block is defined as candidate gene region for each QTL. Heat maps of the differentially expressed genes (DEGs) from transcriptome data of *B. rapa*, which were performed by Chen et al. (2015), the shade of color represent the log_2_ fold changes (inoculated/mock-inoculated) of the DEGs from *B. rapa*. The genes at the left of heat maps are the DEGs in *B. rapa*, and the genes at the right of heat maps are the homoeologous genes of DEGs from *B. rapa* in *B. napus*.

In order to get more evidence to predict candidate genes, the homoeologous genes of DEGs from the transcriptome data after inoculation *P. brassicae* in *B. rapa* were identified in *B. napus* genome (**Figure 4**). For example, there were five SNPs involved in the *MCR-C3* LD block, the candidate gene region was 21.72–21.92 Mb (194.6 Kb) in BnC03, where 44 genes were included, and four of which were the homoeologous genes of four DEGs identified in *B. rapa* (**Figure 4A**). Also, seven SNPs were involved in the LD block for *SCR-C3*, which the candidate region was 57.86–58.10 Mb (339.3 Kb) in BnC03, and 16 genes were predicted in this region. Only one homoeologous gene *BnaC03g68270D* of DEGs corresponding in *B. rapa* was searched (**Figure 4A**). Similarly, 9, 1, 2, 1, 6, 2, and 4 candidate genes obtained from the *B. rapa* homoeologous genes were identified in the candidate regions of *MCR-A4, SCR-A10a, SCR-A10b, SCR-C4a, SCR-C4b, SCR-C6*, and *MCR-C9*, respectively (**Figure 4**; Supplementary Table S2; Supplementary Figure S5). Overall, the 30 homoeologous genes of DEGs from *B. rapa* were likely candidate genes as these nine QTLs (Supplementary Table S2). However, the more evidence needs to be obtained by functional analysis of these genes.

## Discussion

### Physiological Specialization of *P. brassicae* Played an Important Role in the Genetic Complexity of CR

The virulence of pathogen and resistance of host plant also changed along with the continuous game between them. There has been an abundant of research on characterizing the virulence of *P. brassicae* (Xue et al., 2008; Cao et al., 2009), and many races (pathotypes) have been identified by two identification system (Williams classification and European Clubroot Differential). At present, most CR resources identified in *Brassica* were pathotype specific. However, the resistance to single race controlled by single gene was unstable, which was continuously eroded by the pathogen’s adaptation. The above dynamics and instability made the genetic regulation of host plant became more complexity.

Until now, there is no certain conclusion about genetic regulation mode of CR. Genetic analysis showed that both qualitative and quantitative manners existed in the important *Brassica* crops due to the different resistant sources. In *B. rapa*, CR was controlled in a qualitative and quantitative manners both, whereas it was quantitative traits in *B. oleracea* (Piao et al., 2009; Lee et al., 2016). Genetic studies on CR of rapeseed were most based on the sources, of which the resistance derived from *B. rapa* (Zhang et al., 2015). Also, the inheritance of CR in rapeseed was governed by one or two single independent dominant genes (Piao et al., 2009). The difference of genetic regulation mode in CR among different species may be related to the various races. So enhancing the study to physiological race, including developing of new identification system, is the basis to perform intensive study.

### The Results Can Give Some Constructive and Valuable Clues for Resistance Breeding in *B. napus*

There were three and six QTLs were identified in IF and GH, respectively (**Table 2**). But the same QTL between two environments were not detected, which may because that we performed the evaluation at the seedling stage in GH while at the harvest stage in IF. There were maybe different genetic mechanisms between vegetable stage and adult stage in rapeseed. It also gave us an ideal that it can improve the rapeseed CR both in vegetable stage and adult stage by polymerization of all identified CR loci/genes by MAS. The identified CR QTLs were showed additive effect (**Figure 2**), which provided theoretical basis for putting forward the resistant breeding strategy of polymerization of multiple loci, similarly. The SNPs detected in this study could be transformed to PCR markers (Yang et al., 2012), which could prove and consummate the method of screening the resistant germplasms resources and also provide strong and powerful measures for MAS. Meanwhile, we can pyramid different loci that resistant to different pathotypes for breeding to increase the resistant durability (Matsumoto et al., 2012; Tomita et al., 2013).

The results of integration of all the CR QTLs/genes on *B. napus* also gave us revelations that the closely relate species of *B. napus* (e.g., *B. rapa, B. oleracea*, and *B. juncea*) can be used for CR improvement through distant hybridization between these species (**Figure 3**). Therefore, we will have two approaches to improve the rapeseed CR. Firstly, all QTLs/genes which come from *B. napus* and closely relate species can be polymerized by MAS or interspecific hybridization (Yu et al., 2016); Secondly, we can get more resistant *B. rapa* and *B. oleracea* by MAS and phenotypic identification, and then the highly resistance rapeseed can be obtained by artificially synthesized (Diederichsen et al., 2006).

### C-genome Might Have More Potential on CR Improvement than A-genome in *B. napus*

In this study, 6 of 9 identified QTLs were on C-genome, and most of them were novel, which illustrated that the C-genome (*B. oleracea, B. carinata*) had more potential on CR improvement (**Figure 3**). Researchers also found that there were more resistant loci in C-genome than A-genome of *B. napus* in the study of QTL mapping of resistance to *Sclerotinia sclerotiorum* (Wu et al., 2013; Wei et al., 2016). Wang et al. (2014) thought that breeding selection loci of C-genome were more than A-genome variation analysis of rapeseed core germplasms, which further verified that our research results had crucial contribution to resistant breeding in *B. napus*. Therefore, it is necessary for us to expand the resistance sources to discover more resistance genes/loci.

### Integrate Analysis Revealed that the CR Hotspots Existed in *Brassica*

The CR QTL mapping results in *B. rapa* showed that 6 of 11 CR genes/QTLs were on BrA03, which indicated that there were CR hotspots existed in *B. rapa*. Until now, the genome sequences of *B. rapa, B. oleracea*, and *B. napus* have been sequenced which will accelerate their genetic improvements. All reported QTLs/loci information (linkage markers, candidate genes, and positions information) related to CR were collected in these three species (**Figure 3**). Most of the reported CR QTLs/genes were mapped on *B. napus* genome through bioinformatic analyses. The integration results illustrated the CR hotspots also existed in *B. napus*, which were the regions of BnA03 (21–23 Mb), BnC03 (1–2 Mb), and BnC07 (36–39 Mb; **Figure 3**). It was interesting that the top of BnA03 was homoeologous with the top of BnC03, and the bottom of BnA03 was homoeologous with the bottom of BnC07 (Chalhoub et al., 2014). Therefore, the evolution and origin of BnA03, and the genes related to disease-resistance on BnA03 are worth to deeply study. It was a pity that we did not detect any loci in the above hotspot regions of CR. The reason may be that the above hotspot regions were summarized from the population based on bi-parents in *B. rapa*. These hotspots may belong to rare variation sites which were difficult to detect in our natural population, or they were peculiar in *B. rapa*. Another reason maybe was that the previous mapping results were most aimed at the race/pathotype 3, the results of this study was based on the pathotype 4.

### The Functional Annotation of Predicted Candidate Genes Provided Valuable Information for Understanding the Molecular Regulation of CR

So far, there were more than 70 *R* genes cloned from plants (Liu et al., 2007). Most of the cloned *R* genes were NBS-LRR family (Belkhadir et al., 2004; McHale et al., 2006). It was interesting that two *R* genes controlled CR in *B. rapa* were all TIR-NBS-LRR genes, which encoded large and abundant proteins involved in the detection of diverse pathogens. Similarly, the enrichment analysis also found eight pathways which were highly significant with TIR-NBS-LRR genes (Supplementary Figure S4), and there were two TIR-NBS gene clusters in our candidate regions (**Table 3**). NBS-LRR proteins could recognize the specialized pathogen effectors of ETI (also called avirulence proteins). Therefore, TIR-NBS-LRR gene family is most likely to play an important role in the process of the clubroot disease, which needs to deeply study in *Brassica*. Besides that, plant hormones, mainly salicylic acid (SA), jasmonic acid (JA), auxins and cytokinins, also played a role in compatible interactions between *Arabidopsis* and *P. brassicae* (Siemens et al., 2006; Lemarié et al., 2015). It also enlightens us on prediction candidate genes of above pathways and understanding the resistant molecular mechanism. Up to now, only one paper was reported on transcriptome analysis of CR in *Brassica* (Chen et al., 2015). It was a pity that two NILs of *CRb* were used for RNA-Seq in *B. rapa*, not two accessions or varieties. Therefore, these 30 candidate genes identified through the data from RNA-Seq in *B. rapa* just only provided clues for candidate genes prediction and understanding the molecular regulation of CR in *B. napus*. However, it needs to carry out more experiments to obtained more evidence to identify or evidence these candidate genes. For example, the RNA-Seq analysis can be carried out by using of some CR and susceptible lines from the natural population in this study.

## Author Contributions

XW conceived the study. LL and XW designed the experiments. FL performed the genotyping of the association panel. LL, BC, and KX organized the implementation of field trials. LL, YL, FZ, HL, QH, XX, and TZ participated in the phenotyping of CR. LL and FL analyzed the data. LL and XW wrote the paper. YL, FL, JH, and XW participated in the modification of the manuscript. All the authors have read and approved the publication of the manuscript.

## Conflict of Interest Statement

The authors declare that the research was conducted in the absence of any commercial or financial relationships that could be construed as a potential conflict of interest.

## Footnotes

1 http://boxplot.tyerslab.com/

2 http://www.illumina.com

3 http://www.r-bloggers.com
