# Supplementary material for: A Genome-Wide Association Study Reveals New Loci for Resistance to Clubroot Disease in Brassica napus
Source: Front Plant Sci. 2016 Sep 30;7:1483. doi: 10.3389/fpls.2016.01483 (PMC5044777; doi:10.3389/fpls.2016.01483)
Supplement: Supplementary file 1 [file Data_Sheet_1.PDF]

A genome-wide association study reveals new loci for resistance to clubroot disease in *Brassica napus*

Lixia Li, Yujie Luo, Biyun Chen, Kun Xu, Fugui Zhang, Hao Li, Qian Huang, Xin Xiao, Tianyao Zhang, Jihong Hu, Feng Li, and Xiaoming Wu*

Key Laboratory of Biology and Genetic Improvement of Oil Crops, Ministry of Agriculture, Oil Crop Research Institute, Chinese Academy of Agricultural Sciences, Wuhan, Hubei, China

* Corresponding author: Xiaoming Wu. Tel: +86 27 86812906; Fax: +86 27 86812906;
E-mail: wuxm@oilcrops.cn

Running title: GWAS for clubroot resistance

The number of abstract words: 198

The number of manuscript words: 5592

The number of figures: 4

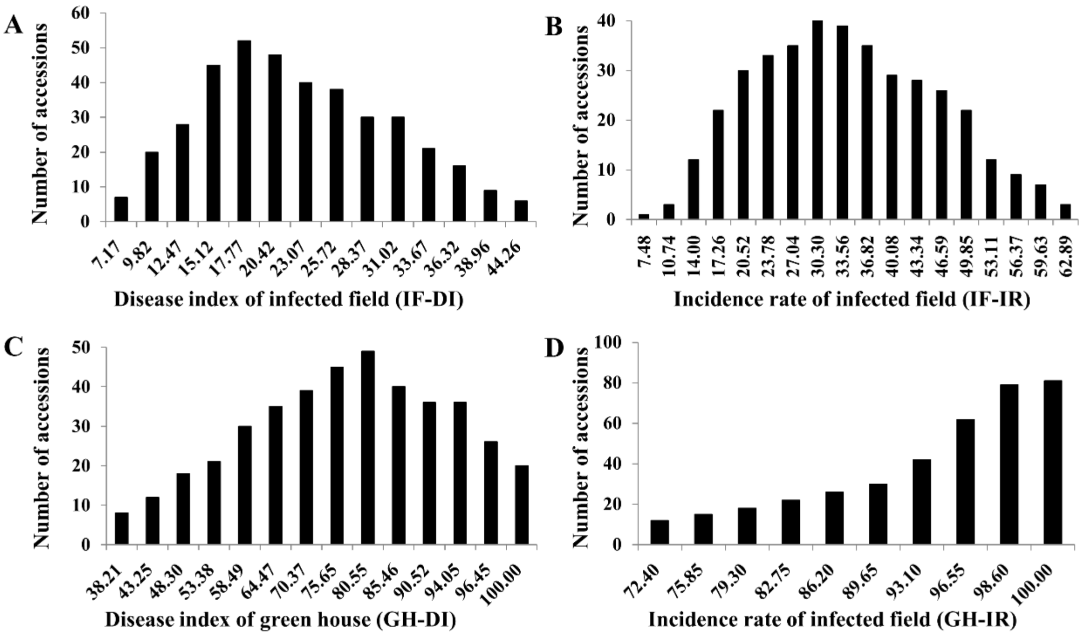

Supplemental Fig. S1 The histograms of the Disease Index (DI) and Incidence Rate (IR) in two environments (IF and GH). (A) The frequency distribution of disease index of infected field. (B) The frequency distribution of incidence rate of infected field. (C) The frequency distribution of disease index of green house. (D) The frequency distribution of incidence rate of greenhouse.

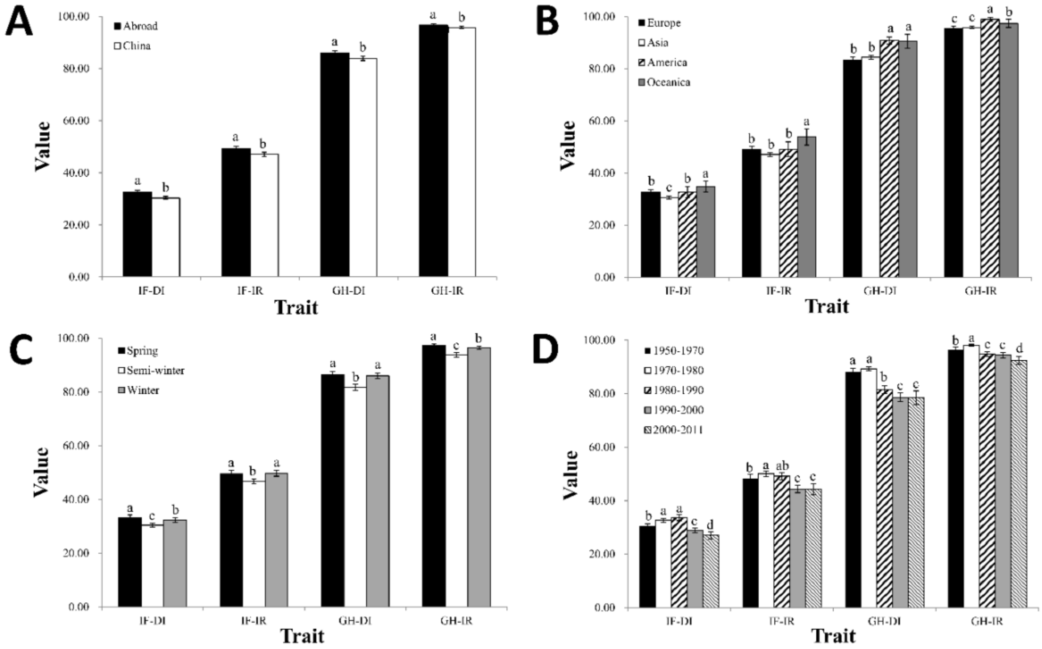

Supplemental Fig. S2 The significant differential analysis based on Disease index (DI) and Incidence Rate (IR) in two environments (IF and GH) among different groups. (A) The significant differential analysis between the groups divided by from China and Abroad. (B) The significant differential analysis among the groups divided by different continents. (C) The significant differential analysis among the groups divided by different ecotypes. (D) The significant differential analysis among the groups divided by different breeding eras.

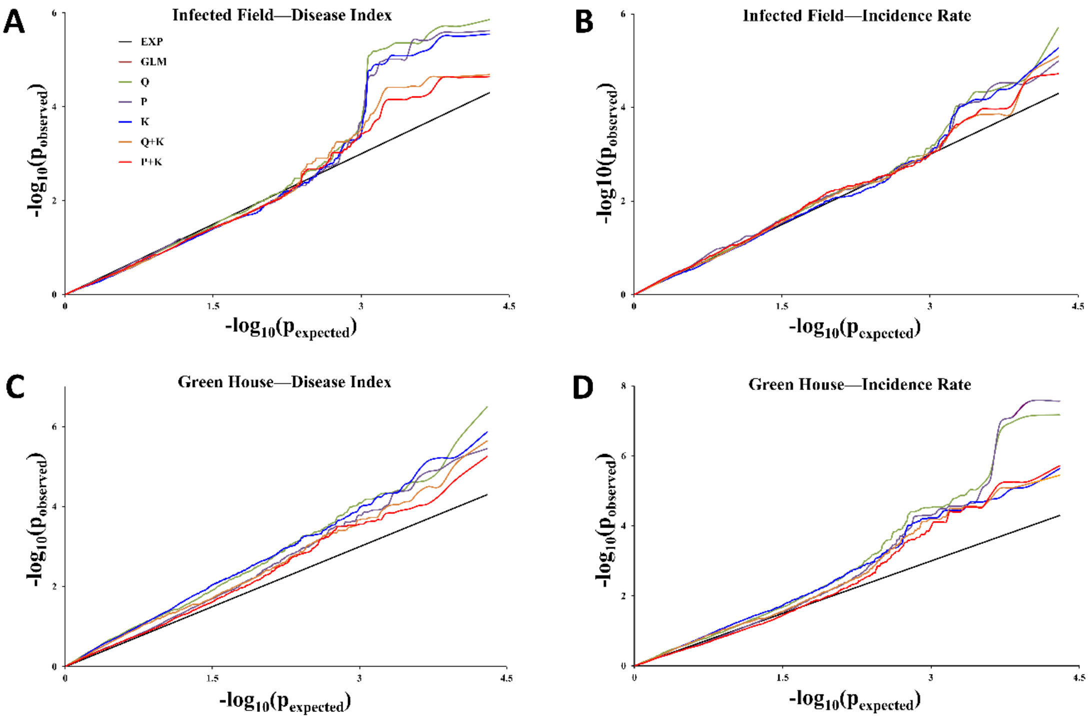

Supplemental Fig. S3 The quantile-quantile (Q-Q) plots for selecting the most appropriate model for genome wide association study (GWAS). (A) The Q-Q plots of different models for IF-DI. (B) The Q-Q plots of different models for IF-IR. (C) The Q-Q plots of different models for GH-DI. (d) The Q-Q plots of different models for GH-IR.

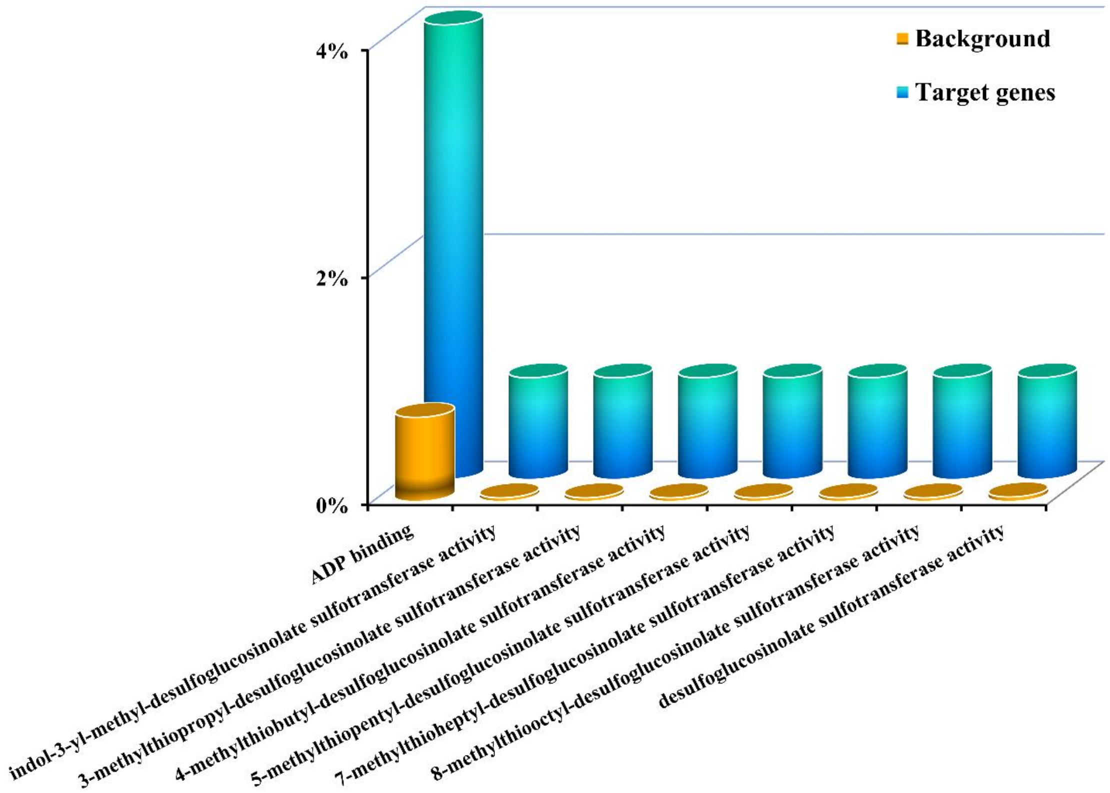

Supplemental Fig. S4 The enrichment analysis of all genes in the candidate regions of the identified loci. The y-axis is the percentage of genes mapped by the term, representing the abundance of the gene ontology (GO) term. The percentage for the input list is calculated by the number of genes mapped to the GO term divided by the number of all genes in the input list. The same calculation was applied to the reference list (background) to generate its percentage.

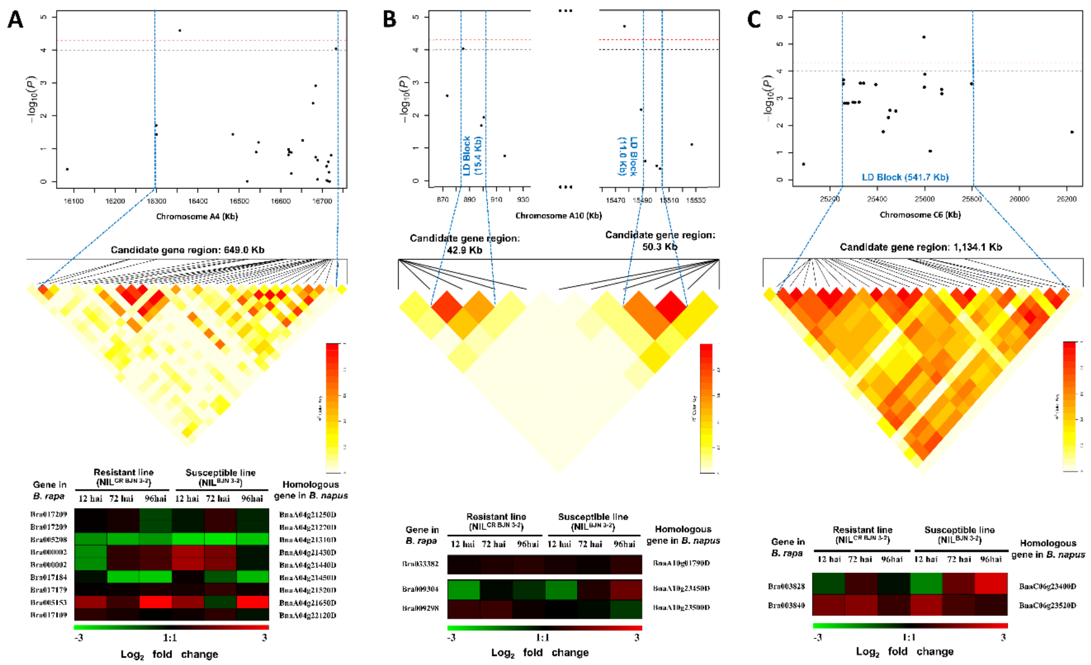

Supplemental Fig. S5 The candidate regions and predicting candidate genes for *MCR-A4*, *SCR-A10a*, *SCR-A10b*, and *SCR-C6*. (A) The candidate regions and predicting candidate genes for *MCR-A4*. (B) The candidate regions and predicting candidate genes for *SCR-A10a* and *SCR-A10b*. (C) The candidate regions and predicting candidate genes for *SCR-C6*. Haplotype block in strong LD (r^2>0.4) with the most significant associated SNPs are shown between the blue dashed line. The chromosome region between the two flanking markers of the LD block is defined as candidate gene region for each QTL. Heat maps of the differentially expressed genes (DEGs) from transcriptome data of *B. rapa*, which were performed by Chen et al. (2016), the shade of colour represent the $\log_2$ fold changes (inoculated/mock-inoculated) of the DEGs from *B. rapa*. The genes at the left of heat maps are the DEGs in *B. rapa*, and the genes at the right of heat maps are the homoeologous genes of DEGs from *B. rapa* in *B. napus*.
