# Supplementary material for: A Genome-Wide Association Study Reveals New Loci for Resistance to Clubroot Disease in Brassica napus
Source: Front Plant Sci. 2016 Sep 30;7:1483. doi: 10.3389/fpls.2016.01483 (PMC5044777; doi:10.3389/fpls.2016.01483)
Supplement: Supplementary file 2 [file Data_Sheet_2.PDF]

A genome-wide association study reveals new loci for resistance on clubroot in *Brassica napus*

Lixia Li, Yujie Luo, Biyun Chen, Kun Xu, Fugui Zhang, Hao Li, Qian Huang, Xin Xiao, Tianyao Zhang, Jihong Hu, Feng Li, and Xiaoming Wu*

Key Laboratory of Biology and Genetic Improvement of Oil Crops, Ministry of Agriculture, Oil Crop Research Institute, Chinese Academy of Agricultural Sciences, Wuhan, Hubei, China

* Corresponding author: Xiaoming Wu. Tel: +86 27 86812906; Fax: +86 27 86812906;
E-mail: wuxm@oilcrops.cn

Running title: GWAS for clubroot resistance

The number of words: 9164

The number of figures: 4

Table S1. The correlation analysis of the traits related to clubroot resistance (CR) between Infected Field (IF) and Greenhouse (GH) of the natural population in *Brassica napus*.

	**IF-DI**	**GH-DI**	**IF-IR**	**GH-IR**
**IF-DI**				
**GH-DI**	0.24^{**}			
**IF-IR**	0.87^{**}	0.22^{**}		
**GH-IR**	0.21^{**}	0.81^{**}	0.19^{**}	

* and ** indicate a significant correlation at the 0.05 probability level and 0.01 probability level, respectively;

IF-DI: Disease index in Infected Field;

IF-IR: Incidence rate (%) in Infected Field;

GH-DI: Disease index in Greenhouse;

GH-IR: Incidence rate (%) in Greenhouse.

Table S2. The candidate genes information of the identified QTLs predicted by using of *B. rapa* transcriptome data.

Locus	Candidate gene region (Mb)	Gene ID	Description
MCR-A4	16.08-16.73	BnaA04g21250D	nuclear factor Y, subunit B8
		BnaA04g21270D	nuclear factor Y, subunit B8
		BnaA04g21310D	pleiotropic drug resistance 5
		BnaA04g21430D	nodulin MtN21/EamA-like transporter family protein
		BnaA04g21440D	nodulin MtN21/EamA-like transporter family protein
		BnaA04g21450D	phenazine biosynthesis PhzC/PhzF family protein
		BnaA04g21520D	ARF-GAP domain 7
		BnaA04g21650D	Bifunctional inhibitor/lipid-transfer protein/seed storage 2S albumin superfamily protein
		BnaA04g22120D	Amino acid dehydrogenase family protein
SCR-A10a	0.87-0.92	BnaA10g01790D	
SCR-A10b	15.48-15.53	BnaA10g23450D	Protein of unknown function (DUF567)
		BnaA10g23500D	Eukaryotic aspartyl protease family protein
MCR-C3	21.72-21.92	BnaC03g35940D	Pectinacetylesterase family protein
		BnaC03g36040D	Transmembrane amino acid transporter family protein
		BnaC03g36150D	Protein kinase protein with tetratricopeptide repeat domain
		BnaC03g36250D	Concanavalin A-like lectin family protein
SCR-C3	57.86-58.10	BnaC03g68270D	Alkaline-phosphatase-like family protein
SCR-C4a	2.46-2.57	BnaC04g03600D	VQ motif-containing protein
SCR-C4b	7.40-8.17	BnaC04g09800D	EID1-like 3
		BnaC04g09840D	Predicted AT-hook DNA-binding family protein
		BnaC04g10090D	zinc finger (CCCH-type) family protein
		BnaC04g10390D	
		BnaC04g10450D	tubulin beta chain 3
		BnaC04g10460D	RNA polymerase I specific transcription initiation factor RRN3 protein
SCR-C6	25.09-26.22	BnaC06g23400D	Calmodulin binding protein-like
		BnaC06g23520D	S-adenosyl-L-methionine-dependent methyltransferases superfamily protein
MCR-C9	41.72-42.80	BnaC09g39140D	beta-amylase 3
		BnaC09g39310D	Protein of Unknown Function (DUF239)
		BnaC09g39740D	copper/zinc superoxide dismutase 3
		BnaC09g40080D	Sodium/calcium exchanger family protein
